# Cervical Spinal Epidural Abscess: Diagnosis, Treatment, and Outcomes: A Case Series and a Literature Review

**DOI:** 10.3390/jcm12134509

**Published:** 2023-07-05

**Authors:** Stamatios A. Papadakis, Margarita-Michaela Ampadiotaki, Dimitrios Pallis, Konstantinos Tsivelekas, Petros Nikolakakos, Labrini Agapitou, George Sapkas

**Affiliations:** 1 B’ Orthopaedic Department, KAT General Hospital of Attica, 14561 Kifissia, Greece; marab.ortho@gmail.com (M.-M.A.); dimitrispallis99@gmail.com (D.P.); tsivelekaskonstantinos@gmail.com (K.T.); petros_nikolakakos@hotmail.com (P.N.); labrini.agapitou@gmail.com (L.A.); 2 Orthopaedic Department, Metropolitan Hospital, 18547 Athens, Greece; gsapkas1@gmail.com

**Keywords:** cervical spinal epidural abscess, surgery, treatment, outcome

## Abstract

Although recent diagnostic and management methods have improved the prognosis of cervical epidural abscesses, morbidity and mortality remain significant. The purpose of our study is to define the clinical presentation of cervical spinal epidural abscess, to determine the early clinical outcome of surgical treatment, and to identify the most effective diagnostic and treatment approaches. Additionally, we analyzed studies regarding cervical epidural abscesses and performed a review of the literature. In this study, four patients with spinal epidural abscess were included. There were three men and one woman with a mean age of 53 years. Three patients presented with motor deficits, and one patient was diagnosed incidentally through spinal imaging. All the patients had fever, and blood cultures were positive. Staphylococcus aureus was the most common organism cultured from abscesses. All patients underwent a surgical procedure, and three patients recovered their normal neurological functions, but one remained with mild neurological disability that was resolved two years postoperatively. The mean follow-up period was 12 months, and no deaths occurred in this series. Furthermore, we identified 85 studies in the literature review and extracted data regarding the diagnosis and management of these patients. The timely detection and effective management of this condition are essential for minimizing its associated morbidity and mortality.

## 1. Introduction

Spinal epidural abscess (SEA) is an infection characterized by the accumulation of purulent material in the space between the dura mater and the osseoligamentous confines of the spinal canal [1,2]. It is an unusual disorder, and in a review carried out by Darouiche et al., the prevalence rate varied from 0.18 to 1.96 per 10,000 admissions in hospitals [3]. Despite recent improvements in the diagnosis and treatment of SEA, the mortality rate is still high, ranging from 4.6% to 31% [4].

Spinal epidural abscess has a peak incidence in the sixth and seventh decades of life [5]. When all large series are considered, male predominance is 2:1 [6]. Predisposing systemic conditions include diabetes mellitus, intravenous drug abuse, renal disease, alcoholism, HIV infection, malignancy, morbid obesity, long-term corticosteroid use, and septicemia [7,8]. Local conditions that predispose an individual to epidural space infection include recent spine trauma, spinal surgery, and intrathecal injection or catheter placement [9].

The responsible pathogens are identified through blood cultures or cultures taken during surgery. Of the microorganisms shown to be causative agents of spinal epidural abscesses, Staphylococcus aureus is the most prevalent [10]. The infection is often caused by Streptococcus species, which are the second most frequently isolated bacteria. Although less common in general, Gram-negative bacilli are frequently isolated from intravenous drug abusers [11]. Mycobacterium tuberculosis, fungal species, and parasitic organisms are rare causes of spinal epidural abscess, especially without associated vertebral osteomyelitis. In some patients, cultures are sterile, and the infecting organism cannot be identified. The mainstay of treatment for spinal epidural abscess is early diagnosis followed by surgical debridement and intravenous antibiotics [12].

Although detection can occur at any level of the spine, epidural abscess in the cervical spine is rare. The incidence of spinal epidural abscess affecting the cervical spine is observed in only 18% to 36% of SEA cases, which is lower than the occurrence in the lumbar or thoracic spine [6]. Despite its lower prevalence, cervical SEA is consistently associated with worse neurological functional outcomes and a higher risk of morbidity and mortality. These findings suggest that the cervical location presents a unique pathology compared to infections in the thoracic or lumbar regions, potentially influenced by factors such as dynamic motion and the presence of the cervical spinal cord [11].

The optimal treatment for cervical epidural abscesses remains controversial. Therefore, the purpose of our study is to define the clinical presentation of cervical spinal epidural abscess in a case series and to determine the early clinical outcome of surgical treatment. Also, we conducted a systematic review of the existing literature related to cervical epidural abscesses.

## 2. Materials and Methods

In this study, four patients with cervical spinal epidural abscess (CSEA) underwent surgical treatment in our department. There were three men and one woman. Their ages varied from 23 to 68 years, and the average age was 53 years.

Three patients presented with motor deficits, and one patient presented incidentally upon spinal imaging. Two patients had involvement of the anterior column of C2–C4, one patient had involvement of C1–C5, and another patient had involvement of C2–C5. All the patients had fever. The time between the appearance of clinical symptoms and surgical treatment was 14 days on average. The median time from admission to surgery was 72 h.

We identified predisposing factors to the development of the infection in two patients. Diabetes mellitus was present in one case and abuse of venous drugs in another.

**Figure 1:**
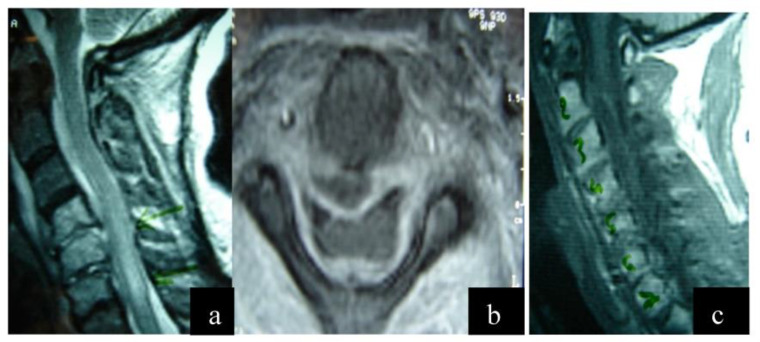
(**a**) Preoperative magnetic resonance imaging (MRI) sequence T2 lateral view. There is a cervical epidural abscess within the spinal canal below the posterior longitudinal ligament extending from C1 to C5, deformation of the signal of the spinal cord due to an inflammatory reaction. (**b**) Preoperative magnetic resonance imaging (MRI) sequence T2 axial view. The presence of a pathological cavity below the posterior longitudinal ligament is observed, causing compression of the thecal sac. (**c**) Preoperative magnetic resonance imaging (MRI) sequence T1 lateral view.

**Figure 2:**
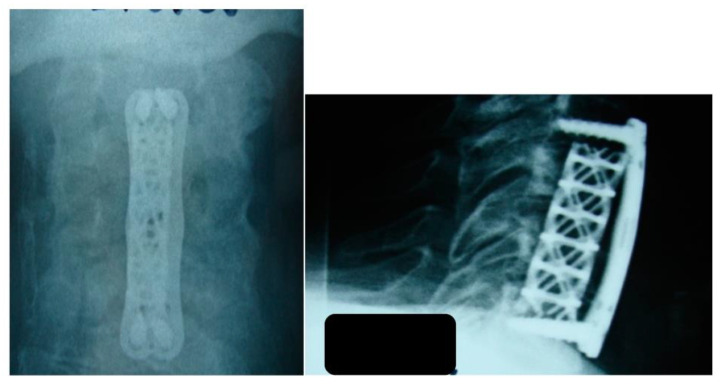
The patient underwent surgical intervention with decompression of the thecal sac. The first procedure was performed using an anterior approach, during which the affected vertebral bodies of C4 and C5 were removed and decompression of the thecal sac was carried out. A titanium cylinder was placed, and anterior stabilization was completed with a plate. Anteroposterior and lateral radiographs.

The infectious agent was identified in all patients through cultures during surgery. Staphylococcus aureus was the predominant germ. Anteroposterior and lateral cervical spine radiographs and Gadolinium-enhanced magnetic resonance imaging (Gd-MRI) were performed in all patients (Figure 1 and Figure 2). In all patients, the lesion was located in the anterior column.

**Figure 3:**
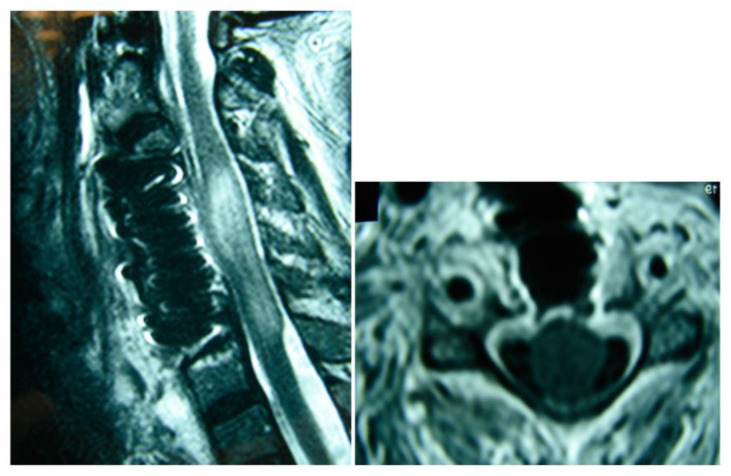
Postoperative magnetic resonance imaging, sagittal and axial views. The presence of a titanium mesh cage and dilation of the spinal cord sac are observed.

**Figure 4:**
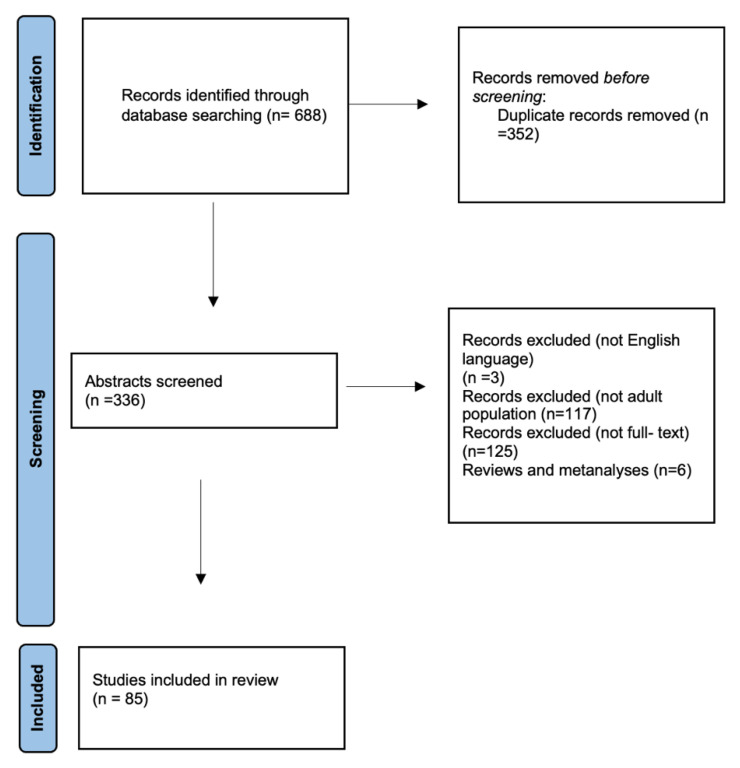
Literature search and flowchart.

All patients underwent decompression under general anesthesia with partial or total corpectomy and fusion using an anterior or posterior approach, debridement, biopsy, and cultures (Figure 3 and Figure 4). Postoperative immobilization with hard cervical orthosis was performed. Intravenous antibiotic therapy was used for 4–6 weeks.

In addition, a literature review was conducted on the PubMed database, using the search terms “cervical epidural abscess” and “surgical treatment” up to December 2022. Two reviewers screened the initial search results and selected studies for review based on the following inclusion criteria: free full text, case reports and case series, English language, adult patients, and studies on humans. Studies were excluded from this review, due to the following exclusion criteria: no English language, full text unavailable, studies on animals, studies on pediatric patients, and inability to determine patients suffering from cervical abscesses from other locations in the same study.

The data that were abstracted from each study were: author, date of publication, total number of patients, gender, age, the level of abscess, pathogen, treatment, outcome, laboratory results, risk factors and previous history, and the presence of spondylodiscitis or an isolated epidural abscess.

## 3. Results

The mean follow-up period of our patients was 12 months (range: 8–18 months). All patients were included in the postoperative evaluation. Three out of the four patients returned to their previous functional status and daily activities fully three months after surgery. In one case, a neurologic deficit was persistent. The patient experienced bilateral upper limb numbness for two years postoperatively, along with muscle weakness graded at 4/5 on the left side and 3.5/5 on the right side. Full recovery was achieved two years postoperatively. Major complications were not observed in any of the patients. There were no deaths in this series, but two cases developed dysphagia, which was resolved without therapy after two weeks (Table 1).

The literature research initially revealed 688 articles related to the term ”cervical epidural abscess”. The full text was available for 211 studies; of those, 208 were written in English. There were 91 referred articles referring to the adult population. We then excluded reviews and metanalyses, and only case series and case reports were included. Thus, a total of 85 studies were included in this review.

The total number of patients included was 209—140 males and 69 females. The mean patient age was 56.2 years old, ranging from 23 to 87. Table 1 demonstrates the patients’ features from each study. Regarding the level of abscess, it was more often observed at C1–C2 and at C5–C6. The most common pathogen was Staphylococcus aureus, observed in 100 cases (30 MRSA and 33 MSSA) (47.9%). Other pathogens that caused cervical abscesses were *Streptococcus* (5.7%), brucellosis (4.7%), *E. coli* (3.3%), *Pseudomonas* (1.9%), *Klebsiella* (1.4%), *Enterococcus*, *Proteus*, and *Mycobacterium tuberculosis*. The patients presented with symptoms including fever, neck pain, numbness, and weakness of the upper limbs. Twenty-five patients (11.9%) had no neurological deficit on admission, although nineteen had quadriparesis (9%). However, most of the patients underwent surgical management, such as corpectomy, fusion, drainage, and decompression, and only 14 patients received conservative treatment (6.6%). The most commonly mentioned risk factors were diabetes mellitus, drug abuse, renal disorder, previous surgical procedures, and dairy product consumption (Table 2).

## 4. Discussion

Spinal epidural abscess is a rare condition that can result in significant morbidity and mortality if not diagnosed and treated in a timely manner [95]. The distinction between acute and chronic disease based on the presence of pyogenic abscess or granulation tissue formation is controversial among authors [96]. The disease can be classified into three phases: acute, subacute, and chronic, and the onset of symptoms usually occurs within hours to days but can also present with a more chronic course over weeks to months [97].

CSEA is most commonly caused by the hematogenous spread of bacteria from a localized infection elsewhere in the body, particularly the skin [97]. In some cases, the source of bacteremia is unknown. Local infections such as spondylitis or paravertebral abscess can also spread to the epidural space, while direct contamination from a penetrating wound or medical procedure can also be a cause of infection. Staphylococcus is the most commonly isolated organism in CSEA, as reported in earlier studies including our review which found it in 47.9% of cases [6]. The onset of symptoms in CSEA may be acute, subacute, or chronic, and can occur within hours to days or over weeks to months. Early diagnosis and prompt treatment are crucial to prevent high morbidity and mortality associated with SEA. 

The incidence of spinal epidural abscess varies depending on the affected segment of the spine. While some authors report the lumbar spine as the most frequent site, others suggest a higher incidence in the thoracic segment. The cervical spine is the least commonly affected, with cases typically associated with spinal osteomyelitis [98]. In a study by Ghobrian et al, C4-C5 was the most common level of involvement in 59 patients with cervical spondylodiscitis who underwent surgical treatment, and they observed that the duration between symptom onset and surgery was a critical factor in the final outcome [57]. Patients with cervical epidural abscess often present with neck pain, fever, difficulty rotating the neck, and neurological deficits. Inflammatory markers such as WBC, ESR, and CRP can support diagnosis. Surgical treatment is strongly indicated in cases of conservative treatment failure, persistent symptoms, presence or deterioration of neurological deficits, spinal instability, abscess larger than 2.5 cm, ischemia or compression, deformities such as kyphosis or scoliosis, and sepsis [99]. In most studies included in the review, surgical treatment and debridement were the preferred options [100,101].

Differential diagnoses of an epidural abscess include spondylosis or degenerative disk syndromes, epidural hematoma, leptomeningeal carcinomatosis, metastatic disease to the spine, spinal cord hemorrhage or infarction, subdural hematoma or empyema, HIV-1-associated myelopathy, tropical myeloneuropathies, vitamin B-12-associated neurological diseases, and alcohol-related neuropathy [102]. Early surgical treatment is recommended over antibiotics alone, according to a study by Alton et al, which compared 62 patients with conservative treatment failure [56]. Tuberculous abscesses have a longer prodrome, frequently lack of leukocytosis and fever, and typically affect younger patients. CT-guided puncture is indicated if conservative treatment is being considered, although there is an additional risk of iatrogenic infection [103]. During the literature review, we found that patients with cervical spinal epidural abscesses due to brucellosis underwent conservative treatment with antibiotics without surgical intervention and achieved favorable outcomes [50,71,74,85].

Magnetic resonance imaging (MRI) is the preferred diagnostic tool for SEA due to its high sensitivity and specificity [7,18,23,26]. The typical MRI findings include a lesion with mass effect and hyper-intense signal on T1-weighted images, which enhances with Gadolinium injection and a nonhomogeneous and hyper-intense signal on T2-weighted images [104].

Surgical intervention is strongly indicated in cases of neural compression, spinal instability, or failure to obtain a satisfactory culture of the infecting organism [11,56]. The procedure typically involves a decompressive laminectomy, drainage of the abscess, and complete debridement of infected tissues. After surgery, patients are usually prescribed antimicrobial therapy for 4 to 6 weeks to prevent recurrence of the infection [11]. Timely diagnosis and management of spinal epidural abscess is critical for improving patient outcomes. Delayed diagnosis and treatment can lead to disease progression, exacerbation of neurological deficits, and increased mortality risk. Research has demonstrated that the duration between symptom onset and surgical intervention is a critical determinant of the final outcome [56]. Therefore, it is crucial to maintain a high level of suspicion regarding SEA in patients with risk factors and to promptly conduct appropriate diagnostic tests and start treatment.

Our patients presented with typical clinical symptoms, including neck pain, fever, and neurological deficits. Diagnosis was confirmed in all cases through magnetic resonance imaging (MRI). From the literature review, it is evident that surgical treatment is preferred in such cases. In two cases, we identified predisposing factors for the development of the infection. One patient had diabetes mellitus, while the other had a history of venous drug abuse.

Early diagnosis and treatment are critical for optimal outcomes in patients with CSEA. By identifying the factors that contribute to early diagnosis and appropriate management, healthcare providers can improve patient outcomes and reduce the risk of complications. This can include implementing screening protocols for high-risk patients, increasing awareness and education among healthcare providers, and promoting timely referral and consultation with specialists. 

## 5. Conclusions

It is important to maintain a high index of suspicion for CSEA in patients with risk factors and relevant symptoms. Early diagnosis is crucial for a better prognosis and the most effective treatment is still immediate surgical drainage of the abscess combined with antibiotics. The limited number of studies in this review highlights the need for further research to establish stronger recommendations for the treatment of CSEA. Overall, timely diagnosis and management are critical in reducing the morbidity and mortality associated with this condition.

## Figures and Tables

**Table 1:** Data of our cases.

Patients	Age	Gender	Level	Micrοorganism	Treatment	Symptoms	Outcome	Risk Factors
1	23	M	C2–C4	Staphylococcus aureus	Debridement and fusion	Fever, pain, numbness, and muscle weakness bilaterally	Full recovery, dysphagia for 2 weeks postop	Abuse of venous drugs
2	68	M	C2–C4	Staphylococcus aureus	Debridement and fusion	Fever, pain, numbness, and muscle weakness bilaterally	Full recovery	Diabetes mellitus
3	56	F	C1–C5	Staphylococcus aureus	Debridement and fusion	Fever, pain, numbness, and muscle weakness bilaterally	Full recovery 2 years post op, muscle weakness	
4	69	M	C2–C5	Staphylococcus aureus	Debridement and fusion	Incidentally upon spinal imaging	Full recovery, dysphagia for 2 weeks postop	

**Table 2:** Literature review of published cases with cervical abscesses. (ps): present study.

Author	Number of Patients	Age	Gender	Level	Micrοorganism	Treatment	Neurological Deficit Initially	Outcome	ESR/WBC/CRP
Frank et al. (1944) [13]	1	43	M	C2	*Staph. aureus*	Hilton’s method	Death from meningitis 15 w post	Death	Raised WBC
Leach et al. (1967) [14]	1	49	F	C1–C2	*Staph. aureus*	Collar, antibiotics	No neurologic deficit	Full recovery 10 months	ESR = 36, WBC = 15
Rimalovski et al. (1968) [15]	1	48	F	C2	*Staph. aureus*	Penicillin, nitrofurantoine, staphcilin 3 months	No neurologic deficit	Death	WBC= 19.9
Ahlback et al. (1970) [16]	2	(1) 44 (2) 43	F M	(1) C1–C2 (2) C1–C2	NA	(1) penicillin, streptomycin, tonsillectomy (2) cloxacillin, C1–C2 fusion	No neurologic deficit	(1) Cervical stiffness (2) Full recovery	(1) ESR = 50, WBC = 8 (2) ESR = 110, WBC = 7.9
Vemireddi (1978) [17]	1	58	M	C1–C2	*Staph. aureus*	Nafcillin, halo, dicloxacillin 3 m	Weakness in upper and lower right extremities	Cervical stiffness	WBC = 7.8, ESR = 74
Venger et al. (1986) [18]	1	29	M	C2	*Staph. aureus*	Halo, nafcillin	No neurologic deficit	Full recovery 6 m	WBC= 18, ESR= 50
Zigler et al. (1987) [19]	5	(1) 62 (2) 66 (3) 67 (4) 56 (5) 72	F M F F M	(1) C1–C2 (2) C1–C2 (3) C1–C2 (4) C1–C2 (5) C1–C2	(1) *Staph. aureus* (2) *Staph. aureus* (3) *Staph. aureus* (4) *Pasteurella multocida* (5) *Staph. aureus*	(1) Oxacillin, posterior cervical fusion C1–C3 (2) Erythromycin, methicillin, halo cast, posterior cervical arthrodesis (3) Cervical traction, transoral biopsy and debridement of axis and atlas, oxacillin (4) Ampicillin, posterior fusion of occiput to axis (5) Oxacillin, posterior atlantoaxial arthrodesis, halo jacket	(1) Weakness in lower extremities (2) No neurological deficits (3) Hyperreflexia (4) Hyperreflexia (5) No neurological deficits	(1) Full recovery (2) Full recovery (3) Full recovery (4) Full recovery (5) Discomfort of the neck secondary to spondylosis	(1) WBC= 7.9 (2) WBC= 7.5, ESR= 108 (3) NA (4) WBC = 39, ESR= 105
Bartels et al. (1990) [20]	1	49	M	C2–C7	*Staph. aureus*	Lateral pharyngotomy to drain a large prevertebral abscess, antibiotics	No neurologic deficit	Full recovery	WBC 13.6
Sebben et al. (1992) [21]	1	59	M	C2–C3	*Staph. aureus*	Decompressive cervicotomy C2–C3		Good recovery	WBC = 8200, TKE = 100, CRP = 35
Ruskin et al. (1992) [22]	1	57	M	C1–C2	*Staph. aureus*, *lactobacillus*	Incision and drainage, imipenem	No neurologic deficit	Full recovery	WBC 17.6, ESR 90
Keogh et al. (1992) [23]	1	41	M	C1–C2	*Staph. aureus*	IV flucloxacillin and fusidic acid; transoral evacuation of extradural pus and excision of eroded odontoid peg; skull traction	No neurologic deficit	Complete resolution at 3 m f/u	WBC 17.9
Azizi et al. (1995) [24]	1	65	M	Clivus-c1	Na	Halo antibiotics	cranial nerve abnormalities	Residual abducens palsy	ESR = 132
Sawada et al. (1996) [25]	1	57	M	C5–C6	*Staph. aureus*	Discectomy	Quadriplegia	Good outcome	WBC = 6300, CRP = 6, ESR = 63
Lam et al. (1996) [26]	1	58	M	C1–C2	*St aureus*	Antibiotics	Bilateral weakness	Full recovery 9 m	ESR = 90, WBC=
Fukutake et al. (1998) [27]	1	74	M	C1–C2	*Streptococcus* pn	Posterior fusion	Numbness of upper extremities	Full recovery 3 m	Esr 127, crp 31, wcc 21.5
Weidau-Pazos et al. (1999) [28]	2	(1) 63 (2) 74	M F	(1) C1–C2 (2) C1–C2	1) *Staph. aureus* 2) NA	(1) transoral decompression, hemilaminectomy (2) transoral decompression, halo, and posterior fusion	Paraparesis	Full recovery	(1) WBC = 13, ESR = 38 (2) WBC = 10, ESR = 85
Anton et al. (1999) [29]	1	75	F	C1–C2	*Strept. viridians*	Decompression, posterior fusion	quadriplegia	Limb weakness	NA
Suchomel et al. (2003) [30]	3	(1) 52 (2) 51 (3) 50	M F M	(1) C1–C2 (2) C1–C2 (3) C1–C2	(1) *Staph. aureus* (2) *Staph. aureus* (3) *Staph. aureus*	Decompression, posterior fusion, antibiotics 3 w	No neurologic deficit All	Full recovery	(1) ESR = 80 (2) WBC, ESR elevated (3) ESR = 90
Haridas et al. (2003) [31]	1	65	M	C2	*Proteus mirabilis*	Transoral decompression, posterior fusion	Upper motor neuron sign both lower extremities, Lhermitte sign (5 d)	Limb paralysis	
Yi et al. (2003) [32]	1	39	M	C5–C6	NA	Laminectomy C5–C6	Decreased upper and lower limb muscle power and bladder dysfunction (10 d)	Full recovery	
Ates et al. (2005) [33]	1	42	F	C3–C5	Brucellosis	Anterior plate and iliac crest graft, doxycycline and rifampicin 3 months	Mild quadriparesis (3 m)	Full recovery	ESR = 80
Burgess et al. (2005) [34]	1		F	C2–C4	MRSA	Laminectomy, dexamethasone, ceftriaxone, and vancomycin (26 h after admission)	Quadriplegia	Death	WBC = 11,400
Moriya et al. (2005) [35]	1	47	M	C3–C5	NA	Cefotaxime and piperacillin	Stiff deep reflexes in lower extremities (10 d)	Good outcome	NA
Paul et al. (2005) [36]	1	54	M	C2–C4	*Pseudomonas aeruginosa*	Decompression, fusion, halo	No neurologic deficit	Neck pain	NA
Kulkarni et al. (2006) [37]	1	56	M	C4–C5	*Serratia marcescens*	Decompression, iliac crest graft	No neurologic Deficit	Neck pain	ESR = 30, CRP = 1.1, WBC = 8
Curry et al. (2007) [38]	1	37	F	C2–C3	NA	Decompression, fusion	No neurologic deficit	Full recovery	WBC = 5.6, ESR = 68
Jeon et al. (2007) [39]	1	72	M	C3–C4	*Eikenella corrodens*	Corpectomy, ciprofloxacin	Right hemiparesis and left hypesthesia	Remaining right hemiparesis and left hypesthesia	CRP = 2, WBC = 12, ESR = 38
Reid et al. (2007) [40]	1	58	M	C1–C2	MRSA	Transoral decompression, posterior fusion	No neurologic deficit	Full recovery	WBC = 14, ESR = 109, CRP = 115
Metcalfe et al. (2009) [41]	1	62	M	C6–C7	*Candida* and *lactobacillus*	C6–C7 partial vertebrectomy, doxycycline, fluconazole	Weakness and pins and needles in both upper limbs, difficulty walking	Full Recovery 17 m	
Hantzidis et al. (2009) [42]	1	65	M	C5–C6	Brucellosis	Cage, anterior plate Doxycycline and streptomycin 3 months	No neurologic deficit	Partial recovery, motor and sensory deficits C6 neurotome	High CRP, IgA, IgG
Fang et al. (2009) [43]	1	31	M	C4–C5	*Staph. aureus*	Corpectomy, fusion, iliac crest graft	No neurologic deficit	Good outcome	9800, 64 CRP = 4.5
Ueda et al. (2009) [44]	1	37	M	C1	*Streptococcus* spp	Antibiotics	No neurologic deficit	Full recovery	WBC = 20, CRP = 4.7
Tamori et al. (2010) [45]	1	80	F	C5–C6	*E. coli*	Decompression, drainage	Brown-Sequard syndrome	Paralysis of right upper limb	WBC = 1.2 CRP = 10
Gezici et al. (2010) [46]	1	(1) 66 (2) 45	M	C4–C5 C5–C7	(1) NA (2) *Staph. aureus*, *Pseudomonas aeruginosa*	(1) Hemilaminectomy, facetectomy (2) Corpectomy, graft	Quadriparesis	Neurologic deficit	(1) Normal (2) WBC = 13, ESR = 136, CRP = 52
Deshmukh (2010) [47]	1	59	F	C2–C3 C7-T1	MRSA	Corpectomy, cervical collar	Quadriparesis	Full recovery	NA
Khoriati et al. (2012) [48]	1	87	M	C2	NA	Occipitocervical fusion	No neurologic deficit	Good recovery	ESR = 91
Ekici et al. (2012) [49]	2	(1) 61 (2) 63	M F	(1) C4–C5 (2) C3–C4	Brucellosis	(1) Decompression and discectomy without fusion, doxycycline, rifampicin for 3 months (2) Decompressive laminectomy, cage, doxycycline, Rifampicin for 3 months	(1) Weakness and hypoesthesia in upper limbs (2) Hypoesthesia in upper limbs	(1) Full recovery (2) Full recovery	(1) WBC = 8.7, CRP = 30.7, ESR = 32 (2) WBC = 7, CRP = 3.8, ESR = 12
Lampropoulos et al. (2012) [50]	1	70	F	C4–C5	Brucellosis	Streptomycin, doxycycline, rifampicin 4 m	No neurological deficits	Recovery	WBC = 6.1, ESR = 80, CRP = 8
Soultanis et al. (2013) [51]	1	53	F	C3–C4	*Enterococcus faecalis*	Decompression–fusion–antibiotics 9 w	Quadriparesis	Improvement	NA
Jensen et al. (2013) [52]	9	(5) 71 (6) 61 (7) 57	F F M	(5) C4–C7 (6) C2–C3 (7) C3–C6	*Strept. anginosus* NA *Staph. aureus*	Spondylodesis C3–C5 Spondylodesis and laminectomy Spondylodesis Antibiotics 3 months	Quadriparesis	Tetraplegia all	NA
Radulovic et al. (2013) [53]	1	53	F	C3–C4	NA	Laminectomies C2–C4	Quadriparesis	Quadriparesis initially, paresis of deltoid finally	WBC-18.7, ESR = 78
O’ neil et al. (2014) [54]	1	64	M	C4–C5	*E. coli*	Discectomy and fusion	Poor balance, motor deficit	Initial poor balance, motor deficits, UTI Eventual improvement	WBC = 24, CRP = 79
Giri et al. (2014) [55]	1	49	M	C5–C6	MRSA	Decompression	No neurologic deficit	NA	ESR = 60, WBC = 2000, CRP = 9
Alton et al. (2015) [56]	62	23 (mean age)	41 M 21 F		MSSA (38.6%) MRSA (32.3%) Streptococcus milleri (4.8%) Unknown (16.3%)	56 treated surgically	23 had neurologic deficit 39 no neurologic deficit	17 remained with neurological deficit	CRP = 168, ESR = 77, WBC = 17
Ghobrial et al. (2015) [57]	40	53 (mean age)	30 M 10 F		MSSA (57.5%) MRSA (12.5%) *Pseudomonas* (5%) *Klebsiella* (2.5%) *E. coli* (2.5%) Negative (12.5%)	NA	NA	6% complication rate	NA
Young et al. (2001) [58]	6	41–74	5 M 1 F	NA	*Staph. aureus*	Anterior corpectomy and fusion	Quadriparesis	4 ambulatory at last/2 quadriparesis	NA
Aranibar et al. (2015) [59]	1	70	F	C1–C2	MRSA	Decompression, posterior fusion occipitocervically	Limb weakness	Limb weakness	NA
Kohlmann et al. (2015) [60]	1	53	F	C2–C5	*E. coli*	Fusion and meropenem	No neurologic deficit	Good outcome	WBC-33, CRP = 163
Ugarriza et al. (2005) [61]	1	55	M	C5–C7	Brucellosis	Decompressive corpectomy and anterior fusion, rifampicin and doxycycline 8 weeks	NA	Full recovery	NA
Oh et al. (2015) [62]	1	44	M	C3–C4	*Strept. viridans*	Ceftriaxone, gentamycin 12 w	No neurologic deficit	Full recovery	WBC = 12, ESR = 23, CRP = 24.9
Zhang et al. (2017) [63]	1	65	F	C6-T8	NA	Imipenem/cilastatin, famciclovir	No neurologic deficit	Full recovery	WBC = 24, ESR = 66, CRP = 193
Lee et al. (2017) [64]	1	49	F	C3–C6	*Staph. aureus*	Laminoplasty	Quadriparesis	Quadriplegia initially, Kyphotic deformity Good outcome	WBC = 23, ESR = 80, CRP = 114
Li et al. (2017) [65]	14	57.7 (mean age)	9 M 5 F	C4–C5(4 patients C5–C6(5) C6–C7(3)		Fusion and ilium bone graft	Quadriparesis		ESR = 63, WBC = 16, CRP = 73
Yang et al. (2017) [66]	1	67	F	C2-T1	*Strept. intermedius*	Vancomycin, decompression	Numbness and weakness of right upper limb and lower limbs	Sensory abnormalities	WBC = 28
Sakaguchi et al. (2017) [67]	1	67	M	C3–C7	*E. coli*	Drainage and antibiotics	NA	Good outcome	WBC = 15, CRP = 28
Kouki et al. (2017) [68]	1	59	M	C3–C5	*Mycobacterium tuberculosis*	Laminectomy	Cervicobrachial neuralgia in the upper extremities and paresthesia (3 m)	NA	
Mc Cann et al. (2018) [69]	1	49	M	C3–C4	*Haemophilus parainfluenzae*	Decompression	No neurological deficit	Good outcome	WBC = 28, CRP = 16
Noori et al. (2018) [70]	1	29	F	C3-T1	*Pseud. aeruginosa*	Laminectomies and cefepime	No neurologic deficit	Good outcome	WBC = 9700
Alyousef et al. (2018) [71]	1	67	M	C5–C7	Brucellosis	Doxycycline, Aminoglycoside, Rifampicin 6 months	No neurologic deficit	Full recovery	WBC= 3.8, ESR = 55, CRP = 152
Thomson et al. (2018) [72]	1	66	F	C1-T4	*Staph. aureus*	Laminectomies, ceftriaxone	Mild quadriparesis	Full recovery	WBC = 20, CRP = 568 mg/dl
Yang et al. (2018) [66]	1	67	F	C5–C6	*Strept. intermedius*	Surgical drainage and irrigation	Weakness in upper and lower extremities	Weakness in upper and lower extremities initially; afterwards, sensory deficit of left leg	WBC = 28
La Fave et al. (2019) [73]	1	45	M	C1–C5	MRSA	C2–C4 laminectomy	Quadriparesis	Persistent limb weakness	WBC = 17.6,
Roushan et al. (2019) [74]	1	43	M	C6–C7	Brucellosis	Rifampicin, doxycycline, gentamycin for 4 months	bilateral hand paresthesia	Full recovery	WBC = 5.8, ESR = 62, CRP = 6
Diyora et al. (2019) [75]	1	30	F	C2–C3	MRSA and *Mycobacterium tuberculosis*	Decompressive laminectomy C2–C3, antibiotics 2 months	Hypotonia of upper and lower limbs	Full recovery	NA
Moustafa et al. (2019) [76]	1	69	M	C6–C7	*E. coli*	Fusion and decompression	Upper- and lower-extremity weakness	Full recovery	ESR = 113, WBC = 24
Zhang et al. (2019) [63]	1	47	M	C5–C6	Βrucellosis	Antibiotics	Incomplete limb paralysis	Good outcome	7600, esr = 86, crp = 55
Lukassen et al. (2019) [77]	1	70	F	C5–C6	*Strept. intermedius*	Corpectomy, fusion	Upper limb paralysis	Good recovery, minor residual hypoesthesia	WBC= 19
Noh et al. (2019) [78]	1	58	F	C5–C6	*Staph. lugdunensis*	C5 corpectomy, Cefazolin, Rifampicin, Cephalexin 8 months	Deltoid weakness, Hoffman, Babinski	Full recovery	ESR= 57, CRP= 1.5
Khan et al. (2020) [79]	1	29	M	C5	Brucellosis	Corpectomy, cage, anterior fusion plate, Rifampicin, doxycycline for 3 months	Numbness of upper limbs	Full Recovery	NA
Sugimoto et al. (2020) [80]	1	87	M	C1–C2	MRSA	Declined surgery, vancomycin 4 w	weakness of extremities	Good outcome (initially)	WBC = 6.4, CRP = 6
Wu et al. (2020) [81]	1	45	F	C4–C7	Anaerobic	meropenem, decompression –fusion	No neurologic deficit	Full recovery	CRP = 94, ESR = 17, WBC = 15
Sati et al. (2021) [82]	1	24	M	C5-T3	*Staph. aureus*	Hemilaminectomy		Wheelchair, urinary catheter	CRP = 132, WBC = 10
Richardson et al. (2021) [83]	1	59	M	C5–C7	*Strept. intermedius*	Vancomycin, meropenem, clindamycin Laminectomy C5–C7	Quadriparesis	Quadriplegia and necrotic fasciitis, death	WBC= 14.8
Gennaro et al. (2021) [84]	1	(1) 56 (2) 55	M M	(1) C4–C6 (2) C5–C7	(1) *Staph. aureus* (2) MRSA	Decompressive laminectomy BOTH	Quadriparesis Quadriparesis	Quadriparesis BOTH	(1) CRP = 37, WBC = 14 (2) WBC = 11.7, CRP = 211
Baghi et al. (2021) [85]	1	22	M	C5–C6	Brucellosis	Doxycycline, aminoglycoside, surgical evaluation, rifampicin for 2 months	No neurologic deficit	Good outcome	WBC = 9.8, CRP= 51
Lewis et al. (2023) [86]	1	55	F	C6–C7	Neisseria	Fusion	No neurologic deficits	Good outcome	
Tomita et al. (2021) [87]	1	79	M	C6–C7	*Klebsiella pneumoniae*	Ct-guided intervertebral drain	Weakness right arm	Good outcome	WBC = 4900, CRP = 3.6
Ntinai et al. (2021) [88]	1	71	M	C2–C7	*Klebsiella pneumoniae*	Drainage, ceftriaxone, ICU	Quadriparesis (2 w) Fever, cardiac arrest	Death	WBC= 21,
Lee et al. (2021) [64]	1	50	M	C3–C5	*Streptococcus agalactiae*	Corpectomy, ampicillin, gentamycin 5 weeks		Full recovery	WBC = 10, CRP = 1.2
Herrera et al. (2022) [89]	1	40	M	C4–C5	MRSA	Vancomycin, metronidazole, Cefepime. Decompression and fusion C4–C7	Quadriparesis	Tetraplegia	ESR = 58, CRP= 4.1 WBC normal
Cao et al. (2022) [90]	1	58	M	C1–C7	*Staph. aureus*	Decompression, ceftriaxone 5 weeks	Weakness in upper and lower limbs	Full recovery 6 m	NA
Abdelraheem et al. (2022) [91]	1	51	F	C5–C7	*Pasteurella multocida*	Cervical corpectomy C6, cage and plate, ceftriaxone	Upper and lower limb weakness	Full recovery	ESR= 135, CRP = 202, WBC = 15
Bara et al. (2022) [92]	1	49	M	C4–C5	*Cutibacterium acnes*	Decompression C4–5, amoxicillin /clavulanic 6 weeks	Lost balance	Full recovery	Elevated
Shin et al. (2022) [93]	1	75	M	C6-T2	*Staph. constellatus*	Decompression corpectomy, discectomy	Paraplegia	Improvement of symptoms, death at 1 year post op	WBC = 15, ESR= 120, CRP= 13
Shafizad et al. (2022) [94]	1	36	M	C5–C6	Brucellosis	C6 corpectomy, cage, anterior fusion	Weakness and hypoesthesia c5–C6	Full recovery	WBC= 14.200, ESR= 33, CRP= 1.3
Sapkas et al. (2023) (ps)	4	53	3Μ 1 F	C1–C5	*Staph. aureus*	Decompression, fusion	Three patients presented with motor deficits, and one incidentally upon spinal imaging, fever	In one case, neurologic deficit remained	

## Data Availability

Not applicable.

## References

[1] Bluman E.M., Palumbo M.A., Lucas P.R. (2004). Spinal Epidural Abscess in Adults. J. Am. Acad. Orthop. Surg..

[2] Savage K., Holtom P.D., Zalavras C.G. (2005). Spinal Epidural Abscess. Early Clinical Outcome in Patients Treated Medically. Clin. Orthop. Relat. Res..

[3] Darouiche R.O., Hamill R.J., Greenberg S.B. (1992). Bacterial spinal epidural abscess. Review of 43 cases and literature survey. Medicine.

[4] Maslen D.R., Jones S.R., Crislip M.A., Bracis R., Dworkin R.J., Flemming J.E. (1993). Spinalepidural abscess: Optimizing patient care. Arch. Intern. Med..

[5] Danner R.L., Hartman B.J. (1987). Update of spinal epidural abscess: 35 cases and review of the literature. Rev. Infect. Dis..

[6] Reihsaus E., Waldbaur H., Seeling W. (2000). Spinal epidural abscess: A meta analysis of 915 patients. Neurosurg. Rev..

[7] Sillevis Smitt P., Tsafka A., van den Bent M., de Bruin H., Hendriks W., Vecht C., Teng-van de Zande F. (1999). Spinal epidural abscess complicating chronic epidural analgesia in 11 cancer patients: Clinical findings and magnetic resonance imaging. J. Neurol..

[8] Still J.M., Abramson R., Law E.J. (1995). Development of an epidural abscess following staphylococcal septicemia in an acutely burned patient: Case report. J. Trauma.

[9] Byers K., Axelrod P., Michael S., Rosen S. (1995). Infections complicating tunneled intraspinal catheter systems used to treat chronic pain. Clin. Infect. Dis..

[10] Veljanoski D., Tonna I., Barlas R., Abdel-Fattah A.R., Almoosawy S.A., Bhatt P. (2023). Spinal infections in the north-east of Scotland: A retrospective analysis. Ann. R. Coll. Surg. Engl..

[11] Epstein N. (2020). Diagnosis, and Treatment of Cervical Epidural Abscess and/or Cervical Vertebral Osteomyelitis with or without Retropharyngeal Abscess; A Review. Surg. Neurol. Int..

[12] Tsantes A.G., Papadopoulos D.V., Vrioni G., Sioutis S., Sapkas G., Benzakour A., Benzakour T., Angelini A., Ruggieri P., Mavrogenis A.F. (2020). World Association Against Infection in Orthopedics And Trauma W A I O T Study Group On Bone And Joint Infection Definitions. Spinal Infections: An Update. Microorganisms.

[13] Al-Hourani K., Al-Aref R., Mesfin A. (2016). Upper Cervical Epidural Abscess in Clinical Practice: Diagnosis and Management. Global Spine J..

[14] Leach R.E., Robert E., Goldstein H. (1967). Howard; Younger, Donna. Osteomyelitis of the Odontoid Process: A Case Report. J. Bone Jt. Surg..

[15] Rimalovski A.B., Aronson S.M. (1968). Abscess of medulla oblongata associated with osteomyelitis of odontoid process. Case report. J. Neurosurg..

[16] Ahlbäck S., Collert S. (1970). Destruction of the Odontoid Process Due to Atlanto-Axial Pyogenic Spondylitis. Acta Radiol..

[17] Vemireddi N. (1978). Osteomyelitis of cervical spine. Orthopaed Rev..

[18] Venger B.H., Musher D.M., Brown E.W., Baskin D.S. (1986). Isolated C-2 osteomyelitis of hematogenous origin: Case report and literature review. Neurosurgery.

[19] Zigler J.E., Bohlman H.H., Robinson R.A., Riley L.H., Dodge L.D. (1987). Pyogenic osteomyelitis of the occiput, the atlas, and the axis. A report of five cases. J. Bone Joint Surg. Am..

[20] Bartels J.W., Brammer R.E. (1990). Cervical osteomyelitis with prevertebral abscess formation. Otolaryngol. Head. Neck Surg..

[21] Sebben A.L., Graells X.S., Benato M.L., Santoro P.G., Kulcheski Á.L. (2017). High cervical spine spondylodiscitis management and literature review. Rev. Assoc. Med. Bras..

[22] Ruskin J., Shapiro S., McCombs M., Greenberg H., Helmer E. (1992). Odontoid osteomyelitis. An unusual presentation of an uncommon disease. West. J. Med..

[23] Keogh S., Crockard A. (1992). Staphylococcal infection of the odontoid peg. Postgrad. Med. J..

[24] Azizi S.A., Fayad P.B., Fulbright R., Giroux M.L., Waxman S.G. (1995). Clivus and cervical spinal osteomyelitis with epidural abscess presenting with multiple cranial neuropathies. Clin. Neurol. Neurosurg..

[25] Sawada M., Iwamura M., Hirata T., Sakai N. (1996). Cervical discitis associated with spinal epidural abscess caused by methicillin-resistant staphylococcus aureus. Neurol. Med. Chir..

[26] Lam C.H., Ethier R., Pokrupa R. (1996). Conservative therapy of atlantoaxial osteomyelitis. A case report. Spine.

[27] Fukutake T., Kitazaki H., Hattori T. (1998). Odontoid osteomyelitis complicating pneumococcal pneumonia. Eur. Neurol..

[28] Wiedau-Pazos M., Curio G., Grüsser C. (1999). Epidural abscess of the cervical spine with osteomyelitis of the odontoid process. Spine.

[29] Anton K., Christoph R., Cornelius F.M. (1999). Osteomyelitis and pathological fracture of the axis. Case illustration. J. Neurosurg..

[30] Suchomel P., Buchvald P., Barsa P., Lukas R., Soukup T. (2003). Pyogenic osteomyelitis of the odontoid process: Single stage decompression and fusion. Spine.

[31] Haridas A., Walsh D.C., Mowle D.H. (2003). Polymicrobial Osteomyelitis of the Odontoid Process with Epidural Abscess: Case Report and Review of Literature. Skull Base.

[32] Yi H.J., Oh S.H., Kwon O.J., Kim H. (2003). Cervical epidural abscess secondary to aorto-duodenal fistula: A case report. J. Korean Med. Sci..

[33] Ates O., Cayli S.R., Koçak A., Kutlu R., Onal R.E., Tekiner A. (2005). Spinal epidural abscess caused by brucellosis. Two case reports. Neurol. Med. Chir..

[34] Burgess C.M., Wolverson A.S., Dale M.T. (2005). Cervical epidural abscess: A rare complication of intravenous cannulation. Anaesthesia.

[35] Moriya M., Kimura T., Yamamoto Y., Abe K., Sakoda S. (2005). Successful treatment of cervical spinal epidural abscess without surgery. Intern. Med..

[36] Paul C.A., Kumar A., Raut V.V., Garhnam A., Kumar N. (2005). Pseudomonas cervical osteomyelitis with retropharyngeal abscess: An unusual complication of otitis media. J. Laryngol. Otol..

[37] Kulkarni A.G., Hee H.T. (2006). Adjacent level discitis after anterior cervical discectomy and fusion (ACDF): A case report. Eur. Spine J..

[38] Curry J.M., Cognetti D.M., Harrop J., Boon M.S., Spiegel J.R. (2007). Cervical discitis and epidural abscess after tonsillectomy. Laryngoscope.

[39] Jeon S.H., Han D.C., Lee S.G., Park H.M., Shin D.J., Lee Y.B. (2007). Eikenella corrodens cervical spinal epidural abscess induced by a fish bone. J. Korean Med. Sci..

[40] Reid P.J., Holman P.J. (2007). Iatrogenic pyogenic osteomyelitis of C1 and C2 treated with transoral decompression and delayed occipitocervical arthrodesis. Case report. J. Neurosurg. Spine.

[41] Metcalfe S., Morgan-Hough C. (2009). Cervical epidural abscess and vertebral osteomyelitis following non-traumatic oesophageal rupture: A case report and discussion. Eur. Spine J..

[42] Hantzidis P., Papadopoulos A., Kalabakos C., Boursinos L., Dimitriou C.G. (2009). Brucella cervical spondylitis complicated by spinal cord compression: A case report. Cases J..

[43] Fang W.K., Chen S.H., Huang D.W., Huang K.C. (2009). Post-traumatic Osteomyelitis with Spinal Epidural Abscess of Cervical Spine in a Young Man with No Predisposing Factor. J. Chin. Med. Assoc..

[44] Ueda Y., Kawahara N., Murakami H., Matsui T., Tomita K. (2009). Pyogenic osteomyelitis of the atlas: A case report. Spine.

[45] Tamori Y., Takahashi T., Suwa H., Ohno K., Nishimoto Y., Nakajima S., Asada M., Kita T., Tsutsumi M. (2010). Cervical epidural abscess presenting with Brown-Sequard syndrome in a patient with type 2 diabetes. Intern. Med..

[46] Gezici A.R., Ergün R. (2010). Cervical epidural abscess in haemodialysis patients by catheter related infection: Report of two cases. J. Korean Med. Sci..

[47] Deshmukh V.R. (2010). Midline trough corpectomies for the evacuation of an extensive ventral cervical and upper thoracic spinal epidural abscess. J. Neurosurg. Spine.

[48] Khoriati A., Kitson J., Deol R.S. (2012). Cervical spinal abscess: An insidious presentation and unusual pathology. Ann. R. Coll. Surg. Engl..

[49] Ekici M.A., Ozbek Z., Gökoğlu A., Menkü A. (2012). Surgical management of cervical spinal epidural abscess caused by Brucella melitensis: Report of two cases and review of the literature. J. Korean Neurosurg. Soc..

[50] Lampropoulos C., Kamposos P., Papaioannou I., Niarou V. (2012). Cervical epidural abscess caused by brucellosis. BMJ Case Rep..

[51] Soultanis K.C., Sakellariou V.I., Starantzis K.A., Stavropoulos N.A., Papagelopoulos P.J. (2013). Insidious Onset of Tetraparesis due to Cervical Epidural Abscess from *Enterococcus faecalis*. Case Rep. Med..

[52] Jensen E.C., Rosted A. (2002). Tuberkuløs spondylit med psoasabsces hos en ung mand uden indvandrerbaggrund [*Tuberculous spondylitis* with a psoas abscess in a young man without an immigrant background]. Ugeskr. Laeger..

[53] Radulovic D., Vujotic L. (2013). Cervical spinal epidural abscess after oesophagoscopy. Eur. Spine J..

[54] O’Neill S.C., Baker J.F., Ellanti P., Synnott K. (2014). Cervical epidural abscess following an *Escherichia coli* urinary tract infection. BMJ Case Rep..

[55] Giri U., Thavalathil B.C., Varghese R. (2014). Vertebral osteomyelitis in an immunosuppressed patient with rheumatoid arthritis. BMJ Case Rep..

[56] Alton T.B., Patel A.R., Bransford R.J., Bellabarba C., Lee M.J., Chapman J.R. (2015). Is there a difference in neurologic outcome in medical versus early operative management of cervical epidural abscesses?. Spine J..

[57] Ghobrial G.M., Beygi S., Viereck M.J., Maulucci C.M., Sharan A., Heller J., Jallo J., Prasad S., Harrop J.S. (2014). Timing in the surgical evacuation of spinal epidural abscesses. Neurosurg. Focus.

[58] Young W.F., Weaver M., Snyder B., Narayan R. (2001). Reversal of tetraplegia in patients with cervical osteomyelitis--epidural abscess using anterior debridement and fusion. Spinal Cord.

[59] Aranibar R.J., Del Monaco D.C., Gonzales P. (2015). Anterior Microscopic Transtubular (MITR) Surgical Approach for Cervical Pyogenic C1-2 Abscess: A Case Report. Int. J. Spine Surg..

[60] Kohlmann R., Nefedev A., Kaase M., Gatermann S.G. (2015). Community-acquired adult *Escherichia coli* meningitis leading to diagnosis of unrecognized retropharyngeal abscess and cervical spondylodiscitis: A case report. BMC Infect. Dis..

[61] Ugarriza L.F., Porras L.F., Lorenzana L.M., Rodríguez-Sánchez J.A., García-Yagüe L.M., Cabezudo J.M. (2005). Brucellar spinal epidural abscesses. Analysis of eleven cases. Br. J. Neurosurg..

[62] Oh J.S., Shim J.J., Lee K.S., Doh J.W. (2015). Cervical epidural abscess: Rare complication of bacterial endocarditis with *Streptococcus viridans*: A case report. Korean J. Spine.

[63] Zhang J.H., Wang Z.L., Wan L. (2017). Cervical epidural analgesia complicated by epidural abscess: A case report and literature review. Medicine.

[64] Lee J.M., Heo S.Y., Kim D.K., Jung J.P., Park C.R., Lee Y.J., Kim G.S. (2021). Quadriplegia after Mitral Valve Replacement in an Infective Endocarditis Patient with Cervical Spine Spondylitis. Korean J. Thorac. Cardiovasc. Surg..

[65] Li H., Chen Z., Yong Z., Li X., Huang Y., Wu D. (2017). Emergency 1-stage anterior approach for cervical spine infection complicated by epidural abscess. Medicine.

[66] Yang C.S., Zhang L.J., Sun Z.H., Yang L., Shi F.D. (2018). Acute prevertebral abscess secondary to intradiscal oxygen-ozone chemonucleolysis for treatment of a cervical disc herniation. J. Int. Med. Res..

[67] Sakaguchi A., Ishimaru N., Ohnishi H., Kawamoto M., Takagi A., Yoshimura S., Kinami S., Sakamoto S. (2017). Retropharyngeal abscess with cervical discitis and vertebral osteomyelitis caused by Escherichia coli in a patient with liver cirrhosis. Infez. Med..

[68] Kouki S., Landolsi M., Ben Lassoued M., Gharsallah I. (2017). Uncommon cause of cervicobrachial neuralgia: Epidural abscess complicating tuberculous arthritis. BMJ Case Rep..

[69] McCann N., Barakat M.F., Schafer F. (2018). An aggressive form of *Haemophilus parainfluenzae* infective endocarditis presenting with limb weakness. BMJ Case Rep..

[70] Noori S.A., Gungor S. (2018). Spinal epidural abscess associated with an epidural catheter in a woman with complex regional pain syndrome and selective IgG3 deficiency: A case report. Medicine.

[71] Alyousef M., Aldoghaither R. (2018). First case of cervical epidural abscess caused by brucellosis in Saudi Arabia: A case report and literature review. Cases.

[72] Thomson C. (2018). Spinal cord compression secondary to epidural abscess: The importance of prompt diagnosis and management. Case Rep..

[73] LaFave J., Bramante R. (2019). Upper Cervical Epidural Abscess Resulting in Respiratory Compromise After Lumbar Steroid Injection. J. Emerg. Med..

[74] Roushan M.R., Ebrahimpour S., Afshar Z.M., Babazadeh A. (2019). Cervical Spine Spondylitis with an Epidural Abscess in a Patient with Brucellosis: A Case Report. J. Crit. Care Med..

[75] Diyora B., Patil S., Bhende B., Patel M., Dhall G., Nayak N. (2019). Concurrent Spinal Epidural Tubercular and Pyogenic Abscess of Cervical Spine without Bony Involvement. J. Neurosci. Rural. Pract..

[76] Moustafa A., Kheireldine R., Khan Z., Alim H., Khan M.S., Alsamman M.A., Youssef E. (2019). Cervical Spinal Osteomyelitis with Epidural Abscess following an Escherichia coli Urinary Tract Infection in an Immunocompetent Host. Case Rep. Infect. Dis..

[77] Lukassen J., Aalbers M.W., Coppes M.H., Groen R. (2019). Cervical spondylodiscitis following cricopharyngeal botulinium toxin injection. Eur. Ann. Otorhinolaryngol. Head. Neck Dis..

[78] Noh T., Zervos T.M., Chen A., Chedid M. (2019). Treatment of a Staphylococcus lugdunensis cervical epidural abscess. BMJ Case Rep..

[79] Khan M.M., Babu R.A., Iqbal J., Batas S.N., Raza A. (2020). Cervical Epidural Abscess due to Brucella Treated with Decompression and Instrumentation: A Case Report and Review of Literature. Asian J. Neurosurg..

[80] Sugimoto H., Hayashi T., Nakadomari S., Sugimoto K. (2020). Delayed diagnosis of an upper cervical epidural abscess masked due to crowned dens syndrome. BMJ Case Rep..

[81] Wu B., He X., Peng B.G. (2020). Pyogenic discitis with an epidural abscess after cervical analgesic discography: A case report. World J. Clin. Cases..

[82] Sati W.O., Haddad M., Anjum S. (2021). A Case of Spinal Epidural Abscess Presenting with Horner Syndrome. Cureus.

[83] Richardson C., Wattenbarger S. (2021). A case report of quadriplegia and acute stroke from tracking retropharyngeal and epidural abscess complicated by necrotizing fasciitis. J. Am. Coll. Emerg. Physicians Open.

[84] Gennaro N., Bonifacio C., Corato M., Milani D., Politi L.S. (2021). Quadriparesis caused by retropharyngeal and epidural abscess in COVID-19 patients. Neurol. Sci..

[85] Baghi M.A., Al-Aani F.K., Rahil A., Ayari B. (2021). Brucellar cervical epidural abscess—A rare cause of neck pain. Cases.

[86] Fox-Lewis A., Luan K., Hopkins C. (2023). Neisseria gonorrhoeae cervical spine epidural abscess requiring spinal decompression and instrumented fusion. J. Infect. Chemother..

[87] Tomita K., Matsumoto T., Kamono M., Miyazaki K., Hasebe T. (2021). CT fluoroscopy-guided percutaneous intervertebral drain insertion for cervical pyogenic spondylodiscitis. Diagn. Interv. Radiol..

[88] Nitinai N., Punpichet M., Nasomsong W. (2021). Fatal Cervical Spinal Epidural Abscess and Spondylodiscitis Complicated with Rhombencephalitis Caused by Klebsiella pneumoniae: A Case Report and Literature Review. Cureus.

[89] Herrera D., Acosta-Rullan J.M., Fox D., Concepcion L., Hughes J. (2022). Quadriplegia from cervical osteomyelodiscitis with vertebral collapse: A case report. Clin. Case Rep..

[90] Cao J., Fang J., Shao X., Shen J., Jiang X. (2022). Case Report: A case of cervical spinal epidural abscess combined with cervical paravertebral soft tissue abscess. Front. Surg..

[91] Abdelraheem M., Mohamed Y., Houlihan E., Murray O. (2022). Treatment of Pasteurella multocida Cervical Epidural Abscess. Cureus..

[92] Bara G.A., Thissen J. (2022). Cervical epidural abscess due to implantation of a spinal cord stimulation lead. Clin. Case Rep..

[93] Shin K.E. (2022). Epidural abscess formation after chemoradiation therapy for esophageal cancer: A case report and literature review. Medicine.

[94] Shafizad M., Ehteshami S., Shojaei H., Jalili Khoshnoud R. (2022). Cervical spine epidural abscess caused by brucellosis: A case report and literature review. Clin. Case Rep..

[95] Tang H.J., Lin H.J., Liu Y.C., Li C.-M. (2002). Spinal Epidural Abscess. Experience with 46 Patients and Evaluation of Prognostic Factors. J. Infect..

[96] Curling D.O., Gower W., McWhorten J.M. (1990). Changing concepts in spinal epidural abscess: A report of 29 cases. Neurosurgery.

[97] Browder J., Meyers R. (1987). Infections of the spinal epidural space: An aspect of vertebral osteomyelitis. Am. J. Sur..

[98] Krauss W.E., McCormick P.C. (1992). Infections of the dural spaces. Neurosurg. Clin. N. Am..

[99] Saeed K., Esposito S., Ascione T., Bassetti M., Bonnet E., Carnelutti A., Chan M., Lye D.C., Cortes N., Dryden M. (2019). International Society of Antimicrobial Chemotherapy (ISAC) Bone and Skin & Soft Tissue Infections Working Group. Hot topics on vertebral osteomyelitis from the International Society of Antimicrobial Chemotherapy. Int. J. Antimicrob. Agents.

[100] Turner A., Zhao L., Gauthier P., Chen S., Roffey D.M., Wai E.K. (2019). Management of cervical spine epidural abscess: A systematic review. Ther. Adv. Infect. Dis..

[101] Bagley C.A., Dudukovich K.J., Wolinsky J.P., Gokaslan Z.L. (2005). Surgical management of lumbosacral spinal epidural abscess. Operat Tech. Neurosurg..

[102] Marais S., Roos I., Mitha A., Mabusha S.J., Patel V., Bhigjee A.I. (2018). Spinal Tuberculosis: Clinicoradiological Findings in 274 Patients. Clin. Infect. Dis..

[103] Inamasu J., Shizu N., Tsutsumi Y., Hirose Y. (2015). Infected epidural hematoma of the lumbar spine associated with invasive pneumococcal disease. Asian J. Neurosurg..

[104] Gupta N., Kadavigere R., Malla S., Bhat S.N., Saravu K. (2023). Differentiating tubercular from pyogenic causes of spine involvement on Magnetic Resonance Imaging. Infez. Med..

